# The Importance of Grey and Qualitative Literature in Developing Domestic Violence and Abuse and Child Maltreatment Core Outcome Sets: A Brief Report

**DOI:** 10.1007/s10896-023-00662-z

**Published:** 2023-10-31

**Authors:** Claire Powell, Siofra Peeren, Ania Ostrowska, Shehzore Adil, Jamie Botevyle, Heather Chesters, Jeanne Wolstencroft, Emma Yapp, Gene Feder, Ruth Gilbert, Emma Howarth

**Affiliations:** 1 Institute of Child Health, UCL, 30 Guilford St, WC1N 1EH London, UK; 2 Service User Research Enterprise, King’s College London, 18 De Crespigny Park, SE5 8AF London, UK; 3 School of Social and Community Medicine, University of Bristol, 39 Whatley Road, Bristol, UK; 4 School of Psychology, UEL, University Way, E16 2RD London, UK

**Keywords:** Domestic Abuse, Child Maltreatment, Outcomes, Grey Literature, Qualitative Literature

## Abstract

**Purpose:**

Core Outcome Sets (COS) are agreed sets of outcomes to be used in all trials that evaluate the effect of interventions. This report considers the added value of including grey and qualitative literature in a study to identify COSs of family-focused interventions for CM and DVA.

**Methods:**

We identified outcomes of interventions for DVA or CM through systematically searching 12 academic databases and 86 organisation websites, leading to the inclusion of 485 full-text reports across 6 reviews. We developed a candidate outcome longlist comprising 347 extracted outcomes.

**Results:**

We identified 87% (282/347) of candidate outcomes from the grey and qualitative literature, and 37% (127/347) from the trial literature. Of the candidate outcomes on the longlist, 22% (75/347) were identified solely from the grey or qualitative literature and 7% (26/347) from trial literature. Three of the eight outcomes in the final core outcome sets may have been missed if grey or qualitative literature had not been searched.

**Conclusions:**

The qualitative and grey literature adds DVA and CM outcomes that are relevant to survivor perspectives but not reported in trials; this had an impact on the final COSs. It is important for COS developers to consider what they may be missing if they do not search the qualitative and grey literature.

## Background

Researcher, clinician, and service user priorities can differ regarding important outcomes for domestic violence and abuse (DVA) and child maltreatment (CM) interventions (Howarth et al., 2015, 2016; MacDonald et al., 2016). DVA and CM are complex clinical, social and public health problems, and the needs of survivors are often overlooked and misunderstood by professionals due to stigma and shame that surrounds experiences of violence and abuse (Mantovani & Allen, 2017; Trevillion et al., 2014). This may result in the success of interventions being measured in ways that are not aligned with survivor conceptions of a good outcome. To address this challenge we developed core outcome sets (COS) for family-focused DVA and CM interventions using consensus methods to arrive at shared priorities among service users, clinicians, and researchers (Howarth et al., 2021; Powell et al., 2022).

COSs are agreed sets of outcomes to be used in all trials that evaluate the effect of interventions. They aim to address both inconsistency in outcome reporting in trials and the gap between researcher priorities, and those of service users and providers concerning how to measure effectiveness (Williamson et al., 2017). The process of developing a COS is two-stage: (1) producing a longlist of candidate outcomes from a range of sources; (2) a consensus process amongst stakeholders to agree on a final set of core outcomes from the longlist (Williamson et al., 2017).

There is extensive and evolving guidance on developing COSs (Williamson et al., 2017). A key element of the process involves conducting a systematic review of existing trials, however the procedure for COS development only advises a review of qualitative studies “if time allows” and there is no mention of reviewing the grey literature^1^ (Williamson et al., 2017, see section 2.7.1.3). This may be because systematic reviews of trial studies are considered the highest level of evidence in the ‘hierarchy of evidence’ (Burns et al., 2011; Costantino et al., 2015; Evans, 2003), even by researchers critical of the narrow focus of randomised trials (Chalmers et al., 2004).

Nevertheless, despite methodological rigour, systematic reviews of trials may miss outcomes of importance to survivors. Researcher and clinician priorities more often guide decisions on the outcomes measured in trial studies (Howarth et al., 2015; Keeley et al., 2016), not least because information about survivors’ own priorities and expectations about effective interventions is more likely to be reported in qualitative and grey literature. Furthermore, survivor-produced knowledge may challenge or conflict with clinician and researcher priorities (Fleischmann, 2009; Keeley et al., 2016; Sweeney, 2009). Thus, prioritising reviews of trials risks excluding outcomes that reflect survivors’ priorities and expectations with regards to interventions; this conflicts with the consensus-based approach of COS development.

In developing our COSs, we incorporated survivor perspectives through reviewing grey and qualitative literature in addition to the trial literature. In this brief report, we consider the value these literatures added to the candidate outcome longlist and final COSs; the full findings are reported elsewhere (Powell et al., 2022).

## Methods

Six rapid reviews were carried out to identify how outcomes were defined, sought or experienced in family-focused domestic violence and abuse (DVA) and child maltreatment (CM) interventions. Our focus was psychosocial interventions for children and families at risk of or with experience of CM or DVA, where DVA was interpersonal abuse between parents/caregivers. The aim of the interventions needed to be to improve child outcomes; this could be through supporting parents or family members or by delivering support to children directly. We reviewed: (1) systematic reviews of DVA intervention trial studies; (2) systematic reviews of CM intervention trial studies; (3) qualitative DVA intervention studies; (4) qualitative CM intervention studies; (5) grey DVA literature; (6) grey CM literature. The protocols were published as part of the full study protocol (Howarth et al., 2021). The purpose of conducting these reviews was to identify candidate outcomes for a longlist to be used in the consensus process.

### Search Strategy and Study Selection

Both the reviews of trial studies and the qualitative studies were designed to build on two previous studies (MacDonald et al., 2016; Howarth et al., 2016), so the searches were run from 2014. Searches for both the trial studies and the qualitative studies were conducted in May 2019.

#### Reviews of Trial Studies

We searched for systematic reviews of DVA and CM intervention trial studies published from 2014 from Medline (Ovid), Embase (Ovid), PsycInfo (Ovid), Cochrane and Web of Science. These included controlled or quasi experimental comparator interventions studies with or without randomisation. We used MeSH terms relating to DVA/CM, the BMJ systematic review strategy and key word terms for DVA/CM, family members and systematic reviews. Search strategies were developed from previous reviews (MacDonald et al., 2016; Howarth et al., 2016) and the BMJ systematic review strategy (BMJ Best Practice, n.d.). (See Howarth et al., 2021 for the full search strategies.)

#### Reviews of Qualitative Studies

We searched ASSIA (ProQuest), CINAHL Plus (EBSCOHost), GoogleScholar (first 100 hits only), PsycInfo (Ovid) and SSCI. We used the same search terms for DVA/CM as the trial studies review with additional search terms for qualitative research.

#### Review of Grey Literature

We searched NICE Evidence Search, Open Grey and 86 relevant organisation websites (selected through expert consultation). Websites were searched manually for relevant documents. Databases were searched using keywords for DVA and CM. (See Powell et al., 2022 for full list of websites and key words.)

### Inclusion Criteria

All studies had to relate to DVA or CM interventions. All searches were completed in Cadima (Kohl et al., 2018) with dual screening of the first 200 title/abstracts for the DVA and CM qualitative and trial reviews as a consistency check. We had 89% agreement on the trial reviews and 86% on the qualitative reviews following pilot screening to test the inclusion criteria. The first 10% of full texts across all reviews were dual screened, with disagreements resolved by discussion prior to screening the remaining full texts by a single researcher. Data extraction of study details and outcomes was carried out in Access (trial studies) or Excel (qualitative and grey literature) by a single researcher with 5% cross-checked by a second researcher.

### Constructing the Candidate Outcome Longlist

We extracted definitions of candidate outcomes (either measured or hoped for) from the full texts of all the included literature to develop a longlist from which to select the COSs through a consensus process. Further candidate outcomes were added from stakeholder workshops and interviews with survivors. We used a team-based iterative approach to deduplicate outcomes, group similar outcomes and develop categories to describe these groups. The origin(s) of each outcome (i.e. whether it came from a literature review, workshop, or interview) was recorded and we noted where the same outcome came from more than one source. We also documented whether a candidate outcome related to children or adults. This process resulted in a longlist of candidate outcomes that was organised into nine broad areas and 39 more specific domains. (For the full list see https://osf.io/yhnfq/). The full process is described in a previous article (Powell et al., 2022).

### Analysis by Type of Literature

We reviewed the longlist of candidate outcomes and analysed the origins of individual outcomes to understand the different contributions of the trial, grey and qualitative literature. We ran analyses in Excel to count and compare the total number of outcomes, and the proportions within each area and domain.

The purpose of this short report is to consider the value that grey and qualitative literature added to the candidate outcome longlist and final COSs. To this end we focus on an examination of outcome domains or areas where the proportion of outcomes from the grey and qualitative literature was particularly high or low: *child sense of self* domain (high; from the area of child health & wellbeing); *safety*^2^ (high) and *violence*^3^ (low), which were areas with related outcomes but contrasting contributions from the literature; *intervention-related outcomes* area (high). These domains and areas were chosen as case studies to demonstrate specific ways that value was added. Rather than providing a comprehensive overview of the findings, we have chosen these domains and areas to provide examples of ways that qualitative and grey literature added value to the longlist and COSs. We also looked at the origins of the two final COSs to understand how the literature reviews affected the outcome of this work. This paper is intended to help future researchers to make an informed decision about whether to include qualitative and grey literature in their own COS studies.

## Findings

Overall, 311 candidate outcomes on the longlist (n = 347) were identified from the rapid reviews. The remaining 36 were identified from the stakeholder workshops and qualitative interviews. The longlist was categorized into nine areas and 39 domains (see https://osf.io/yhnfq/), using pre-existing theoretical frameworks (Belsky, 1993; Firmin, 2017; Heise, 1998). Most outcomes on the longlist of 347 candidate outcomes (282/347, 81%) were identified in the four systematic reviews of the grey or qualitative literature. The two reviews of qualitative DVA/CM studies identified 67% (232/347) of candidate outcomes, the two grey literature reviews identified 50% (173/347), and trial studies identified 37% (127/347). Table 1 shows the proportion of candidate outcomes in the longlist by literature reviewed.

**Table 1:** Percentage of outcomes identified by type of literature reviewed

Type of literature reviewed	Longlist outcomes (n = 347) *n* (%)
DVA/ CM trial studies (2 reviews)	127 (37)
DVA/ CM grey literature (2 reviews)	173 (50)
DVA/ CM qualitative studies (2 reviews)	232 (67)
DVA/CM grey literature or qualitative studies (4 reviews)	282 (81)

If we examine the longlist by area, trials yielded a lower number of candidate outcomes on the longlist compared with the grey and qualitative literature across most outcome areas. The only exception was the *violence, abuse and maltreatment* area where 55% (17/31) of candidate outcomes were identified in the trials, and 39% (12/31) were identified solely in trial studies. See Table 2 for a full breakdown of the proportion of outcomes identified in each area by type of literature.

**Table 2:** Number and percentage of longlist outcomes identified by literature type in each area

Outcome Area	Trial studies (CM & DVA)			Grey literature (CM & DVA)		Qualitative studies (CM & DVA)		
	Outcomes identified *n* (%)	Solely identified* n (%)		Outcomes identified *n* (%)	Solely identified *n* (%)	Outcomes identified *n* (%)	Solely identified *n* (%)	
Child health and well-being (n = 81)	34 (42)	5 (6)		48 (59)	5 (6)	42 (52)	13 (16)	
Caregiver health and well-being (n = 60)	21 (35)	3 (5)		22 (37)	1 (2)	41 (68)	11 (18)	
Parenting (n = 23)	15 (65)	3 (13)		11 (48)	None	19 (83)	4 (17)	
Home (n = 13)	6 (46)	None		7 (54)	1 (8)	10 (77)	3 (23)	
Social Support (n = 13)	2 (15)	None		7 (54)	None	9 (69)	None	
Community (n = 35)	7 (20)	None		26 (74)	3 (9)	23 (66)	4 (11)	
Safety (n = 47)	18 (38)	1 (2)		16 (34)	1 (2)	42 (89)	15 (32)	
Violence (n = 31)	17 (55)	12 (39)		8 (26)	1 (3)	6 (19)	2 (6)	
Intervention-related outcomes (n = 44)	7 (16)	2 (5)		28 (64)	None	40 (91)	11 (25)	
Total (n = 347)	127 (37)	26 (7)		173 (50)	12 (3)	232 (67)	63 (18)	

* The number and percentage of outcomes that were solely identified by that literature for each area

Although many outcomes were identified by more than one type of literature, 22% (75/347) of candidate outcomes on the longlist were identified solely from the grey and qualitative literature, whereas 7% (26/347) of outcomes were identified solely from reports of trial studies. In other words, more than a fifth of our candidate outcomes would have been missed if we had only reviewed trial studies. Of note, 10% (36/347) candidate outcomes on the longlist were not identified in any literature, but instead were identified solely through survivor consultation.

### Child Sense of Self Domain

*Child sense of self* is the largest outcome domain in the broader area of *child health and wellbeing*. Table 3 outlines the 19 outcomes that were captured in this domain along with which literature(s) each outcome was identified by. Fifteen outcomes (79%) were identified in the qualitative literature, 11 (58%) in the grey literature and six (32%) in the trial literature. Importantly, the six outcomes identified in the trial literature were also identified in the other literature, whereas seven outcomes (37%) were identified solely in the grey or qualitative literature.

The outcomes identified in the trial literature were narrower and more easily defined (e.g. resilience, shame, empowerment), whereas outcomes identified in qualitative and grey literature were more detailed and nuanced in conceptualisations of harm, healing, and recovery. Outcomes that were captured by the qualitative and grey literature alone (and which would have otherwise been missed) include ability to assert self, perceptions of self-blame, self-understanding, self-awareness, self-compassion, self-efficacy, self-expression, sense of independence, sense of personal growth and extent to which feel defined by diagnosis. In addition to introducing complexity and nuance, the outcomes identified in qualitative and grey literature reflect conceptualisations of healing and recovery as a long-term and non-linear process (Sinko et al., 2021).

**Table 3:** Overview of outcome origins in the child sense of self domain

	Trial studies (CM & DVA)		Grey literature (CM & DVA)		Qualitative studies (CM & DVA)
Ability to assert self (e.g. say ‘no’)				x	
A sense of empowerment	x		x		
Hope for the future	x		x	x	
Motivation			x	x	
Extent to which feel defined by diagnoses				x	
Perceptions of self-blame (includes guilt)			x		
Resilience	x	x			
Self-compassion				x	
Self-efficacy		x		x	
Self-expression		x		x	
Self-understanding (includes self-awareness)				x	
Self-worth	x	x		x	
Sense of belonging*					
Sense of identity	x	x		x	
Sense of independence				x	
Sense of personal growth				x	
Understand own experiences				x	
Feel in control (includes self-control)		x		x	
Shame	x			x	

*Outcome identified solely from stakeholder workshops and therefore is not presented here

### Comparing the Safety and Violence Areas

*Safety* and *violence* areas were closely linked, with *safety* focusing on the family’s perceptions of safety and knowledge of violence and *violence* covering measures of occurrence, recurrence and re-victimisation of all types of violence and abuse. Most of the 47 *safety* outcomes were identified in the qualitative literature (42 (89%), with 15 (32%) solely identified in the qualitative literature), while most of the 31 *violence* outcomes were identified in the trials (17 (55%), with 12 (39%) solely identified in trial studies). *Violence* was the only outcome area where more outcomes were identified solely in trials than in the grey and/or qualitative literature. See Table 4 below for further details.

**Table 4:** Proportion of outcomes in safety and violence domains by literature type

Domain	Trial studies (CM & DVA)		Grey literature (CM & DVA)		Qualitative studies (CM & DVA)	
	Outcomes identified n (%)	Solely identified n (%)	Outcomes identified n (%)	Solely identified n (%)	Outcomes identified n (%)	Solely identified n (%)
OUTCOME AREA: SAFETY (number of outcomes)						
Safety - includes knowledge and perceptions (n = 7)	3 (43)	None	4 (57)	None	5 (71)	None
Child’s contact with harmful people (n = 2)	1 (50)	1 (50)	1 (50)	None	1 (50)	0
Child’s thoughts and knowledge about their experience (n = 11)	3 (27)	None	4 (36)	1 (9)	9 (81)	3 (27)
Non harming parent’s thoughts and feelings about their child’s experience (n = 12)	4 (33)	None	2 (16)	None	12 (100)	5 (42)
Perpetrator/harmful parent perception of responsibility and understanding of violence and abuse (n = 11)	4 (36)	None	2 (18)	None	11 (100)	6 (55)
Responding after violence and abuse (n = 4)	3 (75)	None	3 (75)	None	4 (100)	1 (25)
**Totals**	**18 (38)**	**1 (2)**	**16 (34)**	**1 (2)**	**42 (89)**	**15 (32)**
OUTCOME AREA: VIOLENCE (number of outcomes)						
Child maltreatment (n = 12)	7 (58)	5 (42)	3 (25)	1 (8)	3 (25)	1 (8)
Domestic violence and abuse between caregivers (n = 15)	8 (53)	7 (47)	3 (20)	None	3 (20)	1 (7)
General violence and abuse (n = 4)	2 (50)	None	2 (50)	None	None	None
**Totals**	**17 (55)**	**12 (39)**	**8 (26)**	**1 (3)**	**6 (19)**	**2 (6)**

Outcomes in the *violence* area focused on measuring actual or risk of harm, whereas outcomes in the *safety* area included survivor perceptions of harm and long-term recovery, such as understanding the dynamics of abuse, understanding consent, attitudes towards gender norms and shifting shame onto perpetrators. One example of the difference in focus between trial and qualitative/grey literature can be seen regarding reproductive coercion. The longlist included two candidate outcomes on reproductive coercion: one in the *violence* area and one in the *safety* area. The item called *reproductive coercion* in the *violence* area related to measuring the presence or absence of reproductive coercion (but not how to define it), whereas the item *understand patterns of abusive behaviour* in the *safety* area conceptualised reproductive coercion as potentially being part of and intersecting with a wider pattern of abuse.

Reproductive coercion has been poorly defined and measured in research, preventing reliable measurement of intervention efficacy (Tarzia & Hegarty, 2021). Indeed, it has been argued that this misunderstanding is rooted in a focus on behaviours (e.g. sexual violence) and outcomes (e.g. unwanted pregnancy), rather than the central role of fear and intent (Tarzia et al., 2020; Tarzia & Hegarty, 2021). Although a full discussion about the definition of reproductive coercion is outside the scope of this report, this example highlights that the outcomes identified by the qualitative and grey literature better reflected emerging definitions and intersections between reproductive coercion and DVA/CM.

### The Area of Intervention-specific Outcomes

Outcome domains included within this broader area of intervention outcomes include: (1) process of intervention delivery; (2) practitioner-related outcomes; (3) intervention adverse effects; (4) experience of intervention. A small proportion of the 44 outcomes in this area was identified in trials (7 (16%)), in contrast with 28 (64%) identified in the grey literature and 40 (91%) in the qualitative literature. Whilst these may be traditionally considered as process outcomes and although they were generally missing in our review of trial studies, these outcomes were highlighted throughout the consensus process by survivors as important to measure.

The two candidate outcomes that related to measuring the adverse effects of interventions, *service harms* and *long-term negative impact of interventions*, were solely identified in the qualitative literature (i.e. 100%). Survivors in the stakeholder workshops and our survivor advisory group underlined the importance of measuring intervention harms, and one of these outcomes was selected for the final CM-COS. Without the qualitative evidence review, this outcome would not have been identified from the literature. The focus on adverse effects from interventions in the qualitative literature and stakeholder engagement fits with critiques of trials as not effectively measuring adverse outcomes (O’Doherty et al., 2014). The importance placed on measuring harm from the intervention may not seem surprising to researchers familiar with one of the key tenets of trauma-informed approaches – that “trauma-uninformed” approaches to care can re-traumatise and cause harm to survivors (Sweeney et al., 2018, p. 322). However, as noted above, even in trials of interventions focused on alleviating the impacts of violence and abuse, this is not well executed.

### The Final COSs

All eight unique outcomes in the COSs were identified in the grey and qualitative literature, whereas only five outcomes were identified in trial studies. Crucially, three outcomes were *solely* identified in the grey and qualitative literature: (1) child has trusted relationships, (2) freedom to go about daily life and (3) adverse effects of interventions. These outcomes were also emphasised by survivors in consensus workshops, demonstrating that the grey and qualitative literature better reflected survivor priorities than current trials.

## Discussion

To summarize, 87% (282/347) of candidate outcomes on the longlist were identified from the grey and qualitative literature, whilst 37% (127/347) were identified from the trial literature. 22% (75/347) of candidate outcomes on the longlist were identified solely from the grey and qualitative literature, whereas only 7% (26/347) of outcomes were identified solely from reports of trial studies. Three of the eight outcomes in the final two core outcome sets were only identified by grey or qualitative literature.

Overall, qualitative and grey literature added value in terms of breadth, depth and relevance of outcomes. When examining the longlist of candidate outcomes identified across the different literatures, we found that: (1) qualitative and grey literature identified more candidate outcomes than the trial literature, (2) all core outcomes and the majority of candidate outcomes would have been identified in the qualitative and grey literature alone, and (3) the outcomes identified by the qualitative and grey literature were more nuanced and orientated to long-term healing and recovery.

By contrast, outcomes identified in the trial literature were narrower, focusing primarily on type of violence perpetrated or clinical diagnoses. The qualitative and grey literature captured aspects of healing and recovery that would otherwise have been missed, such as empowerment, agency, connection and rebuilding trust in self and others. Research shows that these social and longer-term aspects of health and well-being after violence are equally, if not more, important to survivors’ healing and recovery (Sinko et al., 2021). Including qualitative and grey literature in our study therefore ensured the final COS resonated with survivors and addressed holistic needs.

### Involving Survivors as a Research Quality Issue

The main purpose of COSs is to harmonise researcher, clinician, and service user (survivor) priorities to ensure interventions are meaningful for those they are meant to help, and those who are meant to deliver them (Williamson et al., 2017). However, as DVA and CM are complex and often misunderstood clinical, social and public health problems, differences in priorities, perceptions and needs between survivors, clinicians and researchers may be greater than in other areas of health research. Indeed, there is a growing body of evidence showing that the interventions offered to survivors may cause them harm (Sweeney et al., 2018; Scott et al., 2014). Survivors may be harmed through being blamed or disbelieved by professionals (Trevillion et al., 2014; Tarzia et al., 2020) or through approaches that remove power, control and choice (Sweeney et al., 2018, 2019). Representing survivor priorities in research is key not just for maximising intervention effectiveness but, more importantly, for minimizing the potential for interventions to cause direct or indirect harm. Outcomes that related to measuring harm from interventions were solely identified in the qualitative literature, underlining the significant value that including this literature added.

### Minimizing Research Burden for Survivors

Although it is important to involve survivors in research, researchers must avoid adding to the emotional labour that results from expecting survivors to draw on their experiences(Oram et al., 2022; Perôt et al., 2018). Including a review of qualitative and grey literature may reduce emotional labour on survivors in COS studies. Instead of being asked to repeat information that is already captured in the literature, survivors can build upon or unpack the findings of the reviews and thus use their experiential knowledge to develop (and not merely contribute to) findings, ideas, and concepts. Indeed, recent work carried out to develop a Modern Slavery COS involved survivor researchers and extensive review of the qualitative literature (Jannesari et al., in press) to ensure survivors were not consulted in a tokenistic way. In addition to the COSs being used to improve research efficiency in the sector, interventionists might want to use the COSs to assess whether their recovery outcomes are aligned with survivor perspectives.

### Limitations

Reviews were comprehensive but time-limited and focused on the construction of an outcome longlist. We might have arrived at more detailed findings with a more sophisticated meta-synthesis. Our development of the taxonomy, although developed from pre-existing categories (Heise, 1998; Firmin, 2017; Belsky, 1993), was a subjective process – outcomes could have been categorised differently. Had the research team been comprised solely of survivor researchers, it might also have looked different (Gillard et al., 2010). Strengths include the breadth of the reviews and the ability of the reviews to compare the different contributions of the intervention, grey and qualitative literatures.

### Conclusion and Implications

Overall, the qualitative and grey literature added value to the outcomes captured in the longlist and to the COSs via depth, breadth and applicability. We have demonstrated that three out of eight outcomes in the final COSs would have been missed had we looked at the trial literature alone. Therefore, when developing core outcome sets, particularly for complex psychosocial interventions related to trauma, underpinning evidence reviews should not focus solely on trial literature. Failing to include qualitative and grey literature when producing the longlist of candidate outcomes is likely to limit the scope of both the longlist and the final COS.

## Footnotes

1 Grey literature in this study was defined as any relevant practice-based literature not published in academic journals, for example policy documents or charity sector reports.

2 The full name of the Safety area was: Safety & knowledge and understanding of violence and abuse.

3 The full name of the Violence area was: Violence, abuse and maltreatment.
